# Phylogeography and genetic diversity of the copepod family Cyclopidae (Crustacea: Cyclopoida) from freshwater ecosystems of Southeast Nigeria

**DOI:** 10.1186/s12862-020-01608-5

**Published:** 2020-04-21

**Authors:** Yijun Ni, Chike Chukwuenyem Ebido, Elijah Chibueze Odii, Jinhui Wang, Chinemerem Hodges Orakwelu, Francis Chukwuemeka Abonyi, Chinedu Innocent Ngene, Joseph Onyekwere Okoro, Patience Obiageli Ubachukwu, Wei Hu, Mingbo Yin

**Affiliations:** 1 MOE Key Laboratory for Biodiversity Science and Ecological Engineering, School of Life Science, Fudan University, Songhu Road 2005, Shanghai, China; 2 Department of Zoology and Environmental Biology, University of Nigeria, Nsukka, Enugu State 410001 Nigeria

**Keywords:** Cyclopidae, Species diversity, COI, Nigeria

## Abstract

**Background:**

Copepods are key components of aquatic ecosystems and can help regulate the global carbon cycle. Much attention has been paid to the species diversity of copepods worldwide, but the phylogeography and genetic diversity of copepods in Nigeria is unexplored.

**Results:**

Using a mitochondrial cytochrome *c* oxidase subunit I marker, we preformed phylogenetic and phylogeographic analyses for Cyclopidae copepods in Southeast Nigeria. A high species diversity of Cyclopidae in Nigeria: 5 species of *Tropocyclops*, 5 species of *Mesocyclops* and 2 species of *Thermocyclops* from Cyclopidae were identified in 15 populations. Moreover, we detected 18 unique haplotypes, which fell into two distinct clades. Pairwise genetic distances (uncorrected *p*-distances) among the species of Cyclopidae ranged from 0.05 to 0.257. Several species co-existed in the same lake, and some haplotypes were shared among different geographic populations, suggesting a dispersal of Cyclopidae in our sampling region. Finally, we found that the population genetic diversity for each species of Cyclopidae was low in Nigeria.

**Conclusions:**

Our findings explored the species diversity and distribution of copepods within the family Cyclopidae for 15 Nigerian freshwater ecosystems: a high species diversity of Cyclopidae copepods was detected over a small geographic sampling range. Results from this study contribute to a better understanding of copepod diversity of Nigerian freshwater ecosystems.

## Background

Copepods are one of the most taxonomically diverse groups of crustaceans, containing approximately 14,000 described species globally [1]. Copepods can be found in most kinds of aquatic habitats because of their remarkable evolutionary adaptability [1, 2]. They are key components in aquatic ecosystems, playing an important role in food webs [3, 4] and living as endo- or ectoparasites associated with aquatic animals [2, 5, 6]. Many previous studies have shown that copepods are sensitive to climate change [7, 8], because the range of copepods could track the rate of climate change [7]. Copepods can also help regulate the global carbon cycle [9, 10], and they can be used as indicators to natural and anthropogenic environmental stressors by tracing their responses to the elevation of atmospheric CO_2_ levels [11]. Thus, much attention has been paid to the bio-diversity of copepods in aquatic ecosystems [12, 13].

Copepods are the intermediate hosts of the parasitic nematode *Dracunculus medinensis*, which causes a serious Guinea-worm disease in Nigeria and elsewhere [14]. Humans could become infected by drinking unfiltered water containing copepods which are infected with larvae of *D. medinensis*. Therefore, most studies on copepods from Nigeria have focused on their role in the dispersal of the pathogen [15, 16]. Only a few regional biogeographic studies have been conducted on copepods based on morphological species identification [17]. For example, based on the morphology, a previous study showed the occurrence of the genera *Mesocyclops* Sars, 1914 and *Thermocyclops* Kiefer, 1927 in Nigerian freshwater ecosystems: six *Mesocyclops* species and three *Thermocyclops* species were identified [18]. Moreover, it was believed that *M. aspericornis* was one of the most abundant species of *Mesocyclops* in Nigerian waterbodies, and *T. decipiens* was the most abundant species of *Thermocyclops* from Nigeria [18]. However, the identification of different species of copepods solely based on morphology has technical limitations [19], as cryptic species are often detected. Therefore, more discerning methods such as DNA barcoding are needed to investigate copepod taxonomy, especially to recognize morphologically cryptic genetic lineages [20].

DNA barcoding has already been successfully applied to estimate the species/genetic diversity in many zooplankton taxa [21], as it can be used for rapid, accurate, reliable and remote identification of specimens of all metazoan [22]. A fragment of the mitochondrial cytochrome *c* oxidase subunit I (COI) gene has proved to be a useful marker for many biodiversity studies [23, 24], as COI has advantages of being effective for species identification from a wider range of metazoan phyla and possessing a phylogenetic signal which can be used over a wider range of taxonomic levels [22]. The COI marker has been successfully applied to species identification for cladocerans [25, 26]. For example, DNA barcoding was used to identify sibling cryptic species of the *Ceriodaphnia cornuta* species complex from Australia [27] and to examine 61 species of Cladocera, such as *Daphnia*, *Diaphanosoma*, *Ceriodaphnia*, *Moina* and *Alona*, from Mexico and Guatemala [28]. This approach has also been applied to copepods [29]. For instance, a study reported 800 new sequences of 63 marine copepod species by using a COI marker [30]. Elías-Gutierrez et al. [28] examined 21 species of Copepoda from Mexico and Guatemala by applying a COI marker. Using COI is highly advantageous because it can also detect cryptic species, a phenomenon that is very common in copepod assemblages [31]. For example, three genetically divergent but morphologically similar forms of *Hemidiaptomus (Occidodiaptomus) ingens* were detected throughout the distribution range of this species complex [32]. Moreover, *Oithona similis* s.l. was found to be a complex of nine cryptic species instead of a single cosmopolitan species, according to a COI and a nuclear ribosomal 28S genetic marker [20]. Similarly, the nominal species “*Eudiaptomus hadzici*” in the Western Balkans consists of four cryptic species according to a mitochondrial (COI) and a nuclear (nH3) marker [33]. DNA barcoding often reveals differences between allopatric populations. In that situation, it is difficult to decide whether this indicates different genetic lineages or simply geographical intraspecific variation. For instance, several different genetic lineages of *Moina* which were allopatric in a phylogeny were assigned to a single species, because they had similar morphology [34].

The phylogeny of copepods had been widely studied using molecular data. Recently, a comprehensive study from Asia showed a high species diversity of copepods in South Korea [29]. In that study, 133 sequenced individuals represented 94 species belonging to six different orders [29]. Another study has shown that *Sinocalanus tenellus* consists of two very distinct clades in China, suggesting they are parts of a complex of cryptic species [35]. Moreover, Karanovic [36] detected a new species of *Schizopera* from Japan, which was the first member of its genus reported in Japanese freshwater ecosystems, and it had no close relatives from elsewhere in the world. Another study has revised the higher systematics of copepods and proposed the new taxa Canuelloida *ordo. nov.*, Smirnovipinidae *fam. nov.* and Cyclopicinidae *fam. nov.* [37]. Use of molecular data has not been restricted to species-level taxonomy [20], for example, the phylogeography of copepods has been also frequently investigated. They focused on the genealogical lineages of closely related species of copepods and their geographic distributions, by combining the information from phylogenetics, molecular genetics, population genetics, geology, paleontology, demography, ethology and historical biogeography [38]. For instance, two species of copepods (i.e. *Neodiaptomus schmackeri* and *Mongolodiaptomus birulai*) occur in Chinese Taiwan: there was little gene flow among populations for both species [39]. Additionally, four populations of *Leptodiaptomus* cf. *sicilis * in Mexico were found to diverge into 3 distinct phenotypes, and their specialization was further supported by molecular data which showed persistence of a founder effect, limited gene flow, and a pattern of allopatric speciation [40]*.*

There have been no studies on phylogeography and genetic diversity of copepods from Nigeria. In this study, we analyzed 15 copepod populations (out of 32 pools/lakes sampled) from Nigeria. By analyzing sequence variation in the COI gene, we aimed to explore the species diversity and distribution of copepods among these populations. Our expectation was to detect several members of the Cyclopidae, as it is commonly observed worldwide [29, 37]. We also investigated the phylogeography of Cyclopidae in Nigeria.

## Results

### Species and COI genetic diversity

**Fig. 1:**
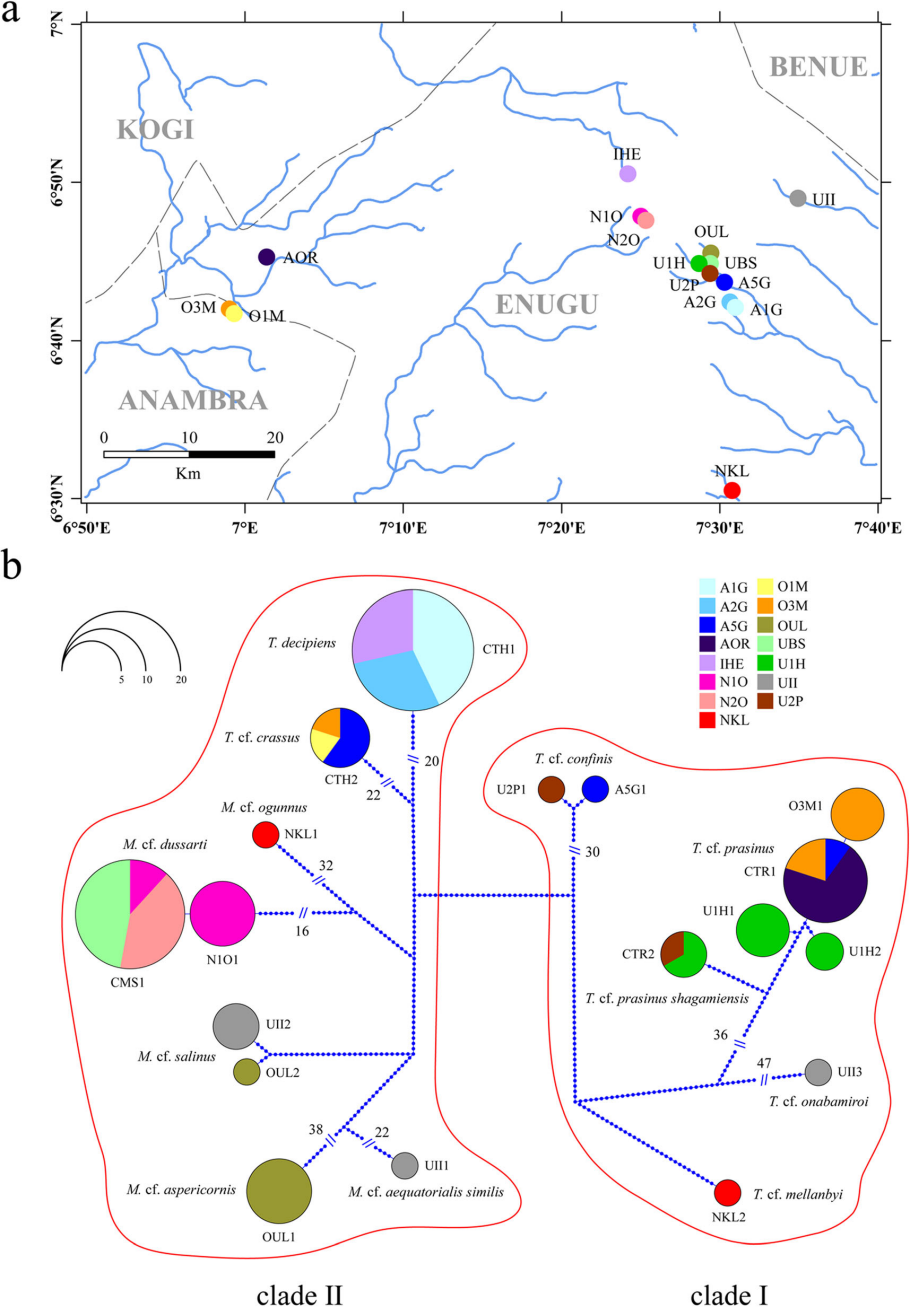
**a** Geographic locations of sampling sites for Cyclopidae in Nigeria. **b** Haplotype network of Cyclopidae from Nigeria, based on the COI gene (544 bp). Each circle represents a unique haplotype and its size reflects the number of individuals expressing that haplotype. Color codes denote geographic location of populations. Portion of circles indicate distribution of haplotypes among different populations. The number of marks on connecting lines indicates the number of mutations between haplotypes. For lake abbreviations see Table 1. The map was obtained from ArcGIS and edited in Adobe Illustrator

**Fig. 2:**
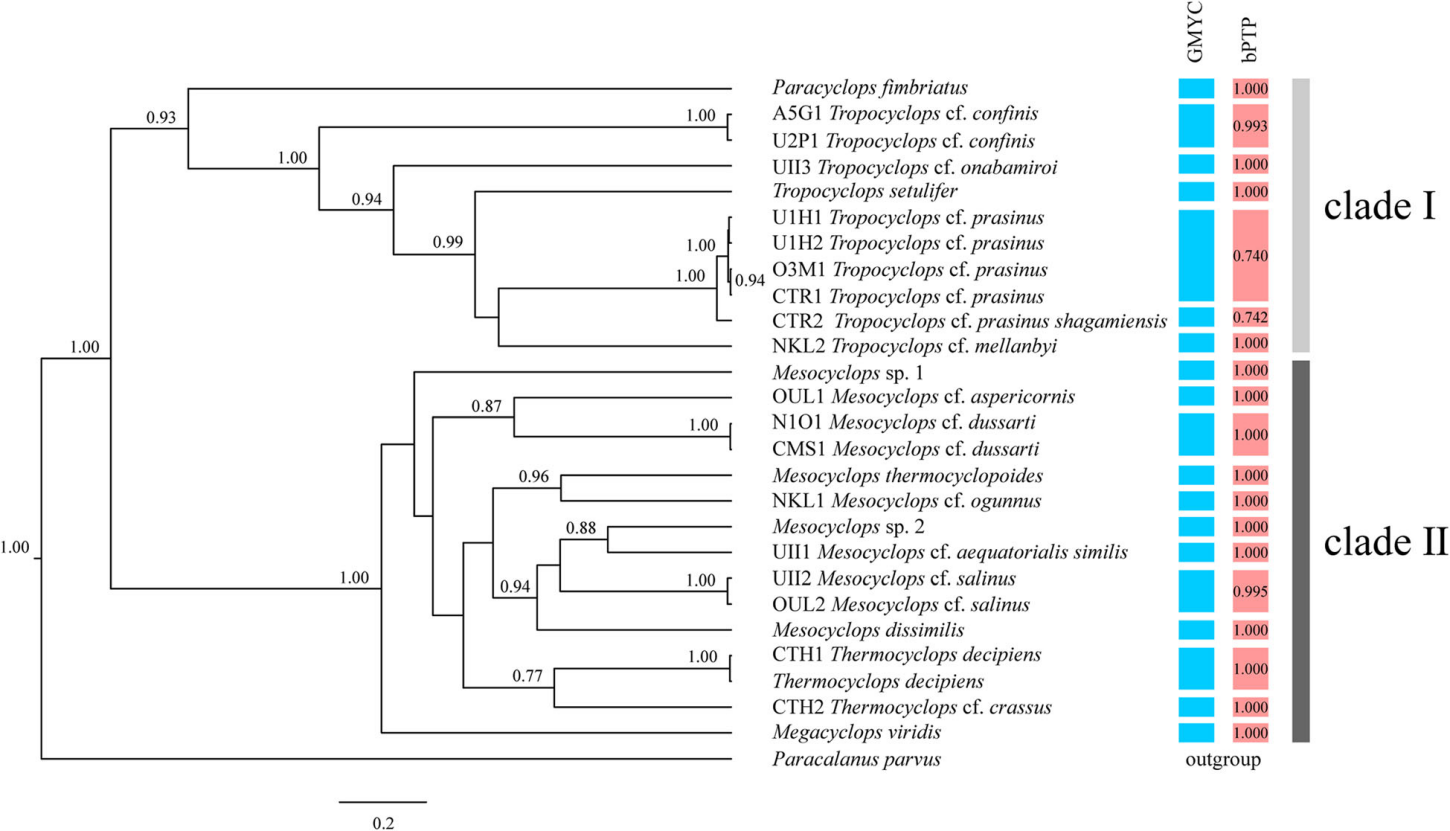
Bayesian phylogenetic tree and species delimitation results for Cyclopidae from Nigeria, based on the COI gene (547 bp). The IDs for shared haplotypes are provided in Table 2; for origin of reference sequence IDs see Table S1. Only posterior probabilities > 0.70 are shown. Species delimitation according to the GMYC and bPTP methods are indicated. For the bPTP method, the statistical support (PP) for species membership is also shown. *Paracalanus parvus* was used as an outgroup

One to 9 specimens of Cyclopidae were sequenced per location, and a total of 88 Cyclopidae COI sequences were successfully obtained from 15 freshwater lakes around Southeast Nigeria, of which 18 unique haplotypes were detected (Tables 1 and 2). None of the COI sequences exhibited characteristics of nuclear pseudogenes (frame shifts or premature stop codons). Two independent species-delimitation methods (i.e. GMYC and bPTP) based on the COI Bayesian tree consistently identified 12 Cyclopidae species from Nigeria: 5 species of *Tropocyclops* (i.e. *T.* cf. *confinis*, *T.* cf. *onabamiroi*, *T.* cf. *prasinus*, *T.* cf. *prasinus shagamiensis* and *T.* cf. *mellanbyi*), 5 species of *Mesocyclops* (i.e. *M.* cf. *aspericornis*, *M.* cf. *dussarti*, *M.* cf. *ogunnus*, *M.* cf. *aequatorialis similis* and *M.* cf. *salinus*) and 2 species of *Thermocyclops* (i.e. *T. decipiens* and *T.* cf. *crassus*; Figs. 1 and 2). Species identified through molecular analyses fell into 2 distinct clades (i.e. clade I: *T.* cf. *confinis*, *T.* cf. *onabamiroi*, *T.* cf. *prasinus*, *T.* cf. *prasinus shagamiensis* and *T.* cf. *mellanbyi*; clade II: *M.* cf. *aspericornis*, *M.* cf. *dussarti*, *M.* cf. *ogunnus*, *M.* cf. *aequatorialis similis, M.* cf. *salinus, T. decipiens* and *T.* cf. *crassus*). Two *Thermocyclops* species were in the same clade as the *Mesocyclops* species. Pairwise genetic distances (uncorrected *p*-distances) based on COI sequence analysis ranged from 0.05 to 0.257 between species (Table 3). For each species, the population haplotype diversity (H_d_) of COI ranged from 0 to 0.533, and the population nucleotide diversity (π) ranged from 0 to 6.86 × 10^− 3^ (Table 2).

**Table 1:** List of localities inhabited by Cyclopidae (name, abbreviation, geographical position), sampling time and water surface temperature

Lake (abbreviation)	Latitude	Longitude	Sampling time	Water surface temperature (°C)
Agu Ekwegbe Pool 1 (A1G)	6.70 °N	7.52 °E	August, 2018	30.3
Agu Ekwegbe Pool 2 (A2G)	6.71 °N	7.51 °E	August, 2018	30.3
Agu Ekwegbe Pool 5 (A5G)	6.73 °N	7.50 °E	August, 2018	30.7
Adanni Opanda Rd. Pool 1 (AOR)	6.75 °N	7.02 °E	September, 2018	29.2
Ihe (IHE)	6.84 °N	7.40 °E	August, 2018	29.7
Nome 1 (N1O)	6.80 °N	7.41 °E	August, 2018	24.7
Nome 2 (N2O)	6.79 °N	7.42 °E	August, 2018	23.9
Nike Lake (NKL)	6.51 °N	7.51 °E	August, 2018	29.4
Omasi Pool 1 (O1M)	6.69 °N	6.99 °E	September, 2018	30.6
Omasi Pool 3 (O3M)	6.70 °N	6.98 °E	September, 2018	29.8
Ogele Ube Lake Opi (OUL)	6.76 °N	7.49 °E	August, 2018	31.1
Uhele Pool Opi (U1H)	6.75 °N	7.48 °E	August, 2018	29.6
Ukwuado Pool 2 Opi (U2P)	6.74 °N	7.49 °E	August, 2018	30.3
Ukwuado Bus Stop Opi Lake (UBS)	6.75 °N	7.49 °E	August, 2018	31.7
Ushuiyi Isusu Ihandiagu (UII)	6.82 °N	7.58 °E	August, 2018	25.9

**Table 2:** Genetic characterization of sequenced individuals of Cyclopidae in each population

Lake (abbreviation)	Mitochondrial gene (COI)							Species
	N_1_	N_2_	Haplotype	H_d_	stdev of H_d_	π	stdev of π	
Agu Ekwegbe Pool 1 (A1G)	9	1	CTH1	0.00	0.00	0.00	0.00	*Thermocyclops decipiens*
Agu Ekwegbe Pool 2 (A2G)	6	1	CTH1	0.00	0.00	0.00	0.00	*Thermocyclops decipiens*
Agu Ekwegbe Pool 5 (A5G)	1	1	A5G1	n.s	n.s	n.s	n.s	*Tropocyclops* cf. *confinis*
	3	1	CTH2	0.00	0.00	0.00	0.00	*Thermocyclops* cf. *crassus*
	1	1	CTR1	n.s	n.s	n.s	n.s	*Tropocyclops* cf. *prasinus*
Adanni Opanda Rd. Pool 1 (AOR)	7	1	CTR1	0.00	0.00	0.00	0.00	*Tropocyclops* cf. *prasinus*
Ihe (IHE)	6	1	CTH1	0.00	0.00	0.00	0.00	*Thermocyclops decipiens*
Nome 1 (N1O)	8	2	N1O1, CMS1	0.429	0.169	7.9 × 10^−4^	3.1 × 10^− 4^	*Mesocyclops* cf. *dussarti*
Nome 2 (N2O)	7	1	CMS1	0.00	0.00	0.00	0.00	*Mesocyclops* cf. *dussarti*
Nike Lake (NKL)	1	1	NKL1	n.s	n.s	n.s	n.s	*Mesocyclops* cf. *ogunnus*
	1	1	NKL2	n.s	n.s	n.s	n.s	*Tropocyclops* cf. *mellanbyi*
Omasi Pool 1 (O1M)	1	1	CTH2	n.s	n.s	n.s	n.s	*Thermocyclops* cf. *crassus*
Omasi Pool 3 (O3M)	1	1	CTH2	n.s	n.s	n.s	n.s	*Thermocyclops* cf. *crassus*
	6	2	CTR1, O3M1	0.533	0.172	9.8 × 10^−4^	3.2 × 10^−4^	*Tropocyclops* cf. *prasinus*
Ogele Ube Lake Opi (OUL)	6	1	OUL1	0.00	0.00	0.00	0.00	*Mesocyclops* cf. *aspericornis*
	1	1	OUL2	n.s	n.s	n.s	n.s	*Mesocyclops* cf. *salinus*
Uhele Pool Opi (U1H)	6	2	U1H1, U1H2	0.533	0.172	6.86 × 10^−3^	2.21 × 10^−3^	*Tropocyclops* cf. *prasinus*
	2	1	CTR2	0.00	0.00	0.00	0.00	*Tropocyclops* cf. *prasinus shagamiensis*
Ukwuado Pool 2 Opi (U2P)	1	1	CTR2	n.s	n.s	n.s	n.s	*Tropocyclops* cf. *prasinus shagamiensis*
	1	1	U2P1	n.s	n.s	n.s	n.s	*Tropocyclops* cf. *confinis*
Ukwuado Bus Stop Opi Lake (UBS)	8	1	CMS1	0.00	0.00	0.00	0.00	*Mesocyclops* cf. *dussarti*
Ushuiyi Isusu Ihandiagu (UII)	1	1	UII1	n.s	n.s	n.s	n.s	*Mesocyclops* cf. *aequatorialis similis*
	3	1	UII2	0.00	0.00	0.00	0.00	*Mesocyclops* cf. *salinus*
	1	1	UII3	n.s	n.s	n.s	n.s	*Tropocyclops* cf. *onabamiroi*

N_1_ is the number of individuals used for COI sequencing, N_2_ is the number of haplotypes, H_d_ is haplotype diversity, stdev of H_d_ is standard deviation of haplotype diversity, π is nucleotide diversity, stdev of π is standard deviation of nucleotide diversity

**Table 3:** Uncorrected pairwise genetic distances (*p*-distances) among species of Cyclopidae copepods based on mitochondrial COI sequence analysis. The number in bracket denote the sample size of each species

	*Tropocyclops* cf. *confinis*	*T.* cf. *onabamiroi*	*T.* cf. *prasinus*	*T.* cf. *prasinus shagamiensis*	*T.* cf. *mellanbyi*	*Mesocyclops* cf. *aspericornis*	*M.* cf. *dussarti*	*M.* cf. *ogunnus*	*M.* cf. *aequatorialis similis*	*M.* cf. *salinus*	*Thermocyclops decipiens*	*T.* cf. *crassus*
*T.* cf. *confinis* (2)												
*T.* cf. *onabamiroi* (1)	0.209											
*T.* cf. *prasinus* (20)	0.224	0.229										
*T.* cf. *prasinus shagamiensis* (3)	0.243	0.230	0.050									
*T.* cf. *mellanbyi* (1)	0.188	0.210	0.193	0.199								
*M.* cf. *aspericornis* (6)	0.211	0.254	0.242	0.243	0.219							
*M.* cf. *dussarti* (23)	0.205	0.255	0.241	0.250	0.205	0.161						
*M.* cf. *ogunnus* (1)	0.209	0.237	0.255	0.254	0.202	0.173	0.152					
*M.* cf. *aequatorialis similis* (1)	0.195	0.257	0.255	0.254	0.204	0.143	0.153	0.147				
*M.* cf. *salinus* (4)	0.210	0.244	0.249	0.247	0.220	0.161	0.154	0.160	0.142			
*T. decipiens* (21)	0.199	0.248	0.233	0.248	0.206	0.165	0.158	0.173	0.149	0.170		
*T.* cf. *crassus* (5)	0.201	0.254	0.245	0.250	0.184	0.176	0.158	0.142	0.131	0.153	0.125	

### Geographic distribution of species

Based on the haplotype network, seven out of 12 species detected through analysis of the COI gene occurred at more than one locality in Nigeria (Fig. 1b). The most frequently occurring species in this study was *T.* cf. *prasinus*, which had 4 haplotypes and was distributed in 4 lakes, including A5G, AOR, O3M and U1H, and one of the 4 haplotypes was shared by 3 lakes (A5G, AOR, O3M). Such a pattern was also observed in species *M.* cf. *dussarti*, which had 2 haplotypes and one of them was shared by 3 lakes (N1O, N2O and UBS) (Fig. 1b). Different Cyclopidae species co-existed in the same lake. For example, three species (i.e. *T.* cf. *confinis*, *T.* cf. *prasinus* and *T.* cf. *crassus*) co-existed in Lake A5G (Fig. 1b). Similarly, *T.* cf. *onabamiroi*, *M.* cf. *aequatorialis similis* and *M.* cf. *salinus* co-existed in Lake UII (Fig. 1b). Moreover, five out of 18 haplotypes were shared by different lakes (Fig. 1b). The most abundant haplotype was CTH1, including 21 specimens shared by A1G, A2G and IHE. This was followed by CMS1, shared by N1O, N2O and UBS, and CTR1, shared by A5G, AOR and O3M (Fig. 1b). Four species (i.e. *T.* cf. *onabamiroi*, *T.* cf. *mellanbyi*, *M.* cf. *ogunnus* and *M.* cf. *aequatorialis similis*) expressed only one haplotype with a single individual (Fig. 1b).

## Discussion

Through analysis of COI sequence variation, we explored the species diversity and distribution of copepods within the family Cyclopidae for 15 Nigerian freshwater ecosystems, the first such study for West Africa. Our results suggested a high species diversity of Cyclopidae copepods over a small geographic sampling range.

High species diversity has already been reported in the copepods from Nigeria [18, 19]. Forty species of Cyclopidae copepods from Nigeria were described based on morphological characteristics in the 1990s [19]. Here, we did not detect any new species based on molecular data; all the 12 species identified in the present study were described in [19]. In agreement with a previous study based on morphology [18], we found that *T. decipiens* was the most abundant species of *Thermocyclops* from Nigeria. However, *M. aspericornis* was recorded as the most abundant species of *Mesocyclops* in Nigerian waterbodies [18], whereas we found that *M.* cf. *dussarti* is the most abundant species of the genus *Mesocyclops*. This inconsistency might be explained by the relatively small sampling region in our study in Southeast Nigeria.

Globally, high levels of species diversity of copepods have also been reported [29, 41]. For example, 53 *Caligus* species were present in Chinese Taiwan, and many more species remained to be discovered from this region [5]. Similarly, thirteen species of Copepoda, including three members of Calanoida (Diaptomidae) and ten members of Cyclopoida (Eucyclopinae and Cyclopinae), were recorded in Chiapas, Mexico [42]. Indeed, high species diversity, even in a relatively small area, has often been observed in copepods [41, 43, 44]. For example, a study identified 43 species that belonged to 11 genera of copepods in Sagami Bay [43]. Another study identified 48 species of copepods in Tolo Harbour, Hong Kong, and *Oithona rigida*, *O. simplex* and *Paracalanus crassirostris* were found to be the most abundant species [44]. Here, we detected 12 species with several species and species complexes across three genera in Nigeria; suggesting a high species diversity of Cyclopidae in Southeast Nigeria.

In agreement with a previous study of Cyclopoida in Nigeria [18], our results showed that the same species could be found in geographically separate populations, which also suggests that there are not extensive and common cryptic species in these sampled lakes. *Thermocyclops decipiens* has also been detected in Antilles, Central America, Columbia, Venezuela, east of the Andes, Brazil [45] and Congo [46], indicating that this species has a wide distribution across continents. In contrast to several copepod species with high genetic divergence over short distances, e.g. *Tigriopus californicus* [47], our data showed genetic similarity of the *T. decipiens* populations from different continents. A similar phenomenon has been detected in some open-ocean copepods which have more obvious dispersal routes. For example, it was found that several mtCOI haplotypes of *Calanus pacificus* were distributed across multiple sampling location from the North Pacific Ocean [48]. By using restriction site-associated DNA sequencing, no significant genetic differentiation was found among *Centropages typicus* samples collected from different NW Atlantic regions with clear connectivity [49]. Zooplankton species often have vast ranges [34, 50]. For example, *Daphnia galeata* has been detected in both China and Europe with some haplotypes shared across large distances [51]. Birds are often regarded as the key vectors for the dispersal of resting eggs of aquatic zooplankton [52], across geographical barriers.

We found that different sibling species of Cyclopidae co-existed in the same Nigerian lake, a common finding in copepods [29]. For example, a study of the genus *Mesocyclops* conducted in Africa reported that *M. major* and *M. ogunnus* often co-existed in the same waterbody [53]. Similarly, another study from Nigeria reported that it was common for up to 3 *Mesocyclops* species co-existed in a single locality [18]. Sympatry provides a possibility for interspecific hybridization, which is believed to be a common phenomenon in zooplankton [54]. Hybridization has often been observed in copepods. For example, hybrids between *Calanus glacialis* and *C. finmarchicus* were detected along the Atlantic and Arctic Canadian coast [55]. Another study also found that hybridization occurred between a female *Neocalanus cristatus* and a male *N. plumchrus*, and was then followed by backcrossing to a *N. plumchrus* individual [56]. Our molecular data indicated paraphyly between *Thermocyclops* and *Mesocyclops*, which might reflect introgression resulting from hybridization [57]. Paraphyly has also been observed in other zooplankton, for example, in the *Daphnia pulex* species complex [58] and *Moina* [34]. Future studies and nuclear markers are needed to investigate gene introgression among the copepod species from Nigeria.

Here, Cyclopidae in Nigeria showed a high species diversity, but for each species, the haplotype diversity and nucleotide diversity were rather low. Consistently, low haplotype and nucleotide diversities were observed in the copepods *Calanus finmarchicus* [59], *C. agulhensis* and *C. sinicus* [60]. Patterns of population genetic diversity could be caused by different evolutionary forces, such as mutation, migration, genetic drift and natural selection. How these evolutionary forces affect the population genetic diversity depends on many factors, including species’ response to environmental changes, and the past and present sizes of the population [59]. Another explanation might be that the structuring of a metapopulation together with founder effects resulted in low population genetic diversity [61, 62]. However, the limited sampling of each species among populations cannot be ruled out as a cause for their low genetic diversity in this study.

## Conclusions

In conclusion, our data revealed a high species diversity of Cyclopidae in Southeast Nigeria: twelve species were detected. Our geographical sampling scale in this study was quite small, and therefore, further studies are called for a comprehensive understanding of species distribution and genetic diversity of Cyclopidae in West Africa.

## Methods

### Sampling

Copepod specimens were collected from 15 freshwater lakes around Southeast Nigeria (Fig. 1a and Table 1). Samples were collected using a 125-μm plankton net hauled vertically through the water column at three different sites per location. Samples collected from different sites in the same location were pooled together and preserved in 95% ethanol. All specimens were identified morphologically according to the morphological description of copepods in Nigeria [17–19, 63], which also worked as taxonomic keys in this paper.

### DNA extraction, PCR amplification and sequencing

Copepods were processed for molecular analyses (Table 2). The cephalosome portion of the prosome was obtained from each individual copepod to avoid DNA contamination from prey items in the gut, using a microscopic tweezer and a sharp blade under the stereomicroscope. Total genomic DNA was extracted from the head using H3 buffer with proteinase K (30 μL), containing 10 mM Tris-HCl, 0.05 M KCl, 0.005% Tween 20, 0.005% NP-40 and 10 mg/ml proteinase K (MERCK, Germany). Samples were incubated overnight at 55 °C in a water-bath with mild shaking. The proteinase K was irreversibly denatured after a 12-min incubation at 95 °C. The homogenate was centrifuged briefly and stored at 4 °C before use. A 680 base-pair fragment of the COI gene was amplified using the consensus primer pair (forward: 5′- GGT CAA CAA ATC ATA AAG ATA TTG G − 3′; reverse: 5′- TAA ACT TCA GGG TGA CCA AAA AAT CA -3′ [64];). Polymerase chain reaction (PCR) was carried out in a total volume of 20 μL, consisting of 2 μL 10 X PCR buffer (10 mM Tris-HCl, pH 8.3, 5 mM MgCl_2_, 50 mM KCl), 2 μL 2.5 mM of each dNTP, 1 μL 0.5 μM of each primer, 11.6 μL water, 2 units of Taq DNA polymerase (SuperTherm DNA polymerase, *Taq HS* from TAKARA BIO INC., California, USA) and 2 μL of genomic DNA. The PCR thermocycle protocol was as follows: denaturation at 94 °C for 1 min, then 40 cycles of 1 min at 94 °C, 1.5 min at 40 °C and 1.5 min at 72 °C; followed by a final elongation at 72 °C for 6 min. The success of amplification was checked using agarose gel electrophoresis. Afterwards, the PCR products were purified (High Pure PCR Product Purification Kit, Roche Diagnostics) and sequenced in the forward direction on an ABI PRISM 3730 DNA capillary sequencer by Invitrogen Trading Co., Ltd. (China). Chromatograms were checked for ambiguous base calling and errors in base calling were corrected using MEGA 6 [65], and the Phred quality scores of the sequences were examined with Chromas Lite Version 2.1 (Technelysium Pty. Ltd., South Brisbane, Australia). Sequences with double peaks or noise were re-sequenced in the reverse direction, and only chromatograms of high quality were applied to the following genetic analyses. All newly obtained sequences were submitted to GenBank under accession numbers MN990471-MN990488.

### Sequence alignment and genetic diversity

We identified unique haplotypes in DnaSP 5.10 [66]. MUSCLE [67] implemented in MEGA 6 was used to align the sequences that were subsequently translated into amino acids to examine the presence of stop codons. Afterwards, all haplotypes were aligned, together with 8 reference sequences obtained from GenBank (Table S1), using the Clustal W algorithm [68] in MEGA 6. Then, all the sequences were timed to a uniform length of 544 bp in MEGA 6. For each species, the number of haplotypes (N_2_), haplotype diversity (H_d_) and nucleotide diversity (π) per population (populations with sample size less than 3 were excluded) were calculated in DnaSP 5.10.

### Phylogenetic analyses

The test of Xia et al. [69] implemented in DAMBE 5 [70] was used to inspect potential loss of phylogenetic signal resulting from substitution saturation at the COI locus. A phylogenetic tree was then constructed using the Bayesian method in BEAST 2 [71], with a tree sampled every 1000 generations among 10,000,000, a burn-in of 25%, and the final 10,000 trees summarized using TreeAnnotator. The best-fitting substitution model was GTR + G + I according to the corrected Akaike Information Criterion in jModeltest v. 2.1.7 [72]. We applied a strict molecular clock and set other tree priors to their default values. Tracer v1.6 [73] was applied to ensure that enough generations were computed. *Paracalanus parvus*, a member of the Calanoida phylogenetically close to Cyclopoida, was used as an outgroup.

### Species identification and phylogeographic analyses

To test the hypothesis that the Cyclopidae in Nigeria contains high biodiversity, two independent species delimitation methods were applied: the general mixed Yule coalescent model (GMYC [74]) and Poisson tree processes methods (PTP [75]). The GMYC model is a likelihood-based method using an ultrametric tree to delimit species by fitting within- and between-species branching models to reconstruct gene trees. We performed the GMYC modeling using the “splits” package [76] in R 2.15 [77] and conducted the PTP calculations on the bPTP webserver (http://species.h-its.org/ptp/), with 100,000 Markov Chain Monte Carlo (MCMC) generations, thinning set to 100, burnin at 25% and a Bayesian search performed. The input phylogenetic tree was generated using BEAST 2 (see above). A network of COI haplotypes was then constructed to visualize genetic relationships among populations using Haploviewer [78]. The maximum likelihood tree inferred with MEGA 6 using the best model GTR + G + I (by jModeltest v. 2.1.7) was applied as input. Uncorrected pairwise genetic distances between species were calculated in MEGA 6 based on COI.

## Supplementary information

**Additional file 1: Table S1.** List of reference COI sequences of Cyclopidae (from South Korea, Brazil and China) and the outgroup used in this study.

## Data Availability

All the sequencing data are available via NCBI (under accession numbers MN990471-MN990488).

## Abbreviations

COI: Mitochondrial cytochrome *c* oxidase subunit I gene
PCR: Polymerase chain reaction
bp: Base pairs
GMYC: The general mixed Yule coalescent model
PTP: Poisson tree processes methods
