# Supplementary material for: Phylogeography and genetic diversity of the copepod family Cyclopidae (Crustacea: Cyclopoida) from freshwater ecosystems of Southeast Nigeria
Source: BMC Evol Biol. 2020 Apr 21;20:45. doi: 10.1186/s12862-020-01608-5 (PMC7171763; doi:10.1186/s12862-020-01608-5)
Supplement: Supplementary file 1 — Additional file 1: Table S1. List of reference COI sequences of Cyclopidae (from South Korea, Brazil and China) and the outgroup used in this study. [file 12862_2020_1608_MOESM1_ESM.docx]

Table S1. List of reference COI sequences of Cyclopidae (from South Korea, Brazil and China) and the outgroup used in this study.

| Region | Species name | GenBank ID | Reference |
| --- | --- | --- | --- |
| South Korea | *Paracalanus parvus* | KR048951.1 | Baek et al., (2016) |
| South Korea | *Megacyclops viridis* | KR048972.1 | Baek et al., (2016) |
| South Korea | *Mesocyclops dissimilis* | KR048974.1 | Baek et al., (2016) |
| South Korea | *Paracyclops fimbriatus* | KR048978.1 | Baek et al., (2016) |
| South Korea | *Tropocyclops setulifer* | KR048979.1 | Baek et al., (2016) |
| Brazil | *Mesocyclops* sp. | MF443203.1 | Domingos et al., direct submission |
| Brazil | *Thermocyclops decipiens* | MF443204.1 | Domingos et al., direct submission |
| China | *Mesocyclops* sp. | KJ020570.1 | Young et al., direct submission |
| China | *Mesocyclops thermocyclopoides* | KJ020572.1 | Young et al., direct submission |
